# Annotations

**Published:** 1893-06-17

**Authors:** 


### ANNOTATIONS.

**Medical Protection and Medical Defence.**

To distinguish between things that differ is the work of science: to unite things between which there ought to be no quarrel is the work of statesmanship. At the present moment the medical profession has a Medical Protection Society and a Medical Defence Union. The chief *raison d'etre* of the Protection Society since its formation has been to defend the profession against the Defence Union. But the Defence Union has now purged itself of its offences and offenders, and stands before the world clean and of a sound mind. Its finances also are approximately sound, although the failure to capitalize the subscriptions of life members has been a very unbusiness-like performance, and may yet land the Union in serious difficulties. The Protection Society, on the other hand, has been judiciously financed from the first, and is exceedingly sound and solvent. These two societies are doing the same kind of work, and the competition between the two has acted as a stimulant to both, and good results have followed. Nevertheless, there are serious economic objections against the multiplication of societies having the same object. Besides, half-a-dozen small and weak societies invite contempt, where one strong one might command respect and confidence. Is it possible for these two societies to be united? We think it is, and as desirable as it is possible. The Defence Union can bring a large experience into the contract, and even if the experience includes partial failure and some mistakes, that also is valuable as a warning. The Protection Society can bring young and active blood, a distinguished president, capable officials, and a strenuous and united Council. What hinders that an amalgamation should take place? We know of nothing. The question seems to be well worthy of the consideration of the Councils of the two Societies.

**A Childish Quarrel at Oxford.**

OXFORD furnishes us at the present moment with a foolish little illustration of the dictum that "men are but children of a larger growth." The Oxford city coroner is a person "robed in a little brief authority," and he appears to be somewhat magnificently conscious of the fact. The Committee of the Radcliffe Infirmary, on the other hand, seem to lack the capacity for dealing in a dignified manner with a person who is determined to make himself ridiculous at their expense. This is the *casus belli*. The coroner claims the power to order that the dead bodies of those persons who die in the Radcliffe Infirmary, upon which inquests are necessary, shall be removed from the infirmary to the mortuary for the purposes of judicial inquiry. The Infirmary Committee, on their part, seem to doubt whether the coroner has really the legal right he claims, and, in any case, they are of opinion that there can be no real necessity for removing bodies from the infirmary to the mortuary under ordinary circumstances. There is probably no doubt that the law gives the coroner the powers he asserts, and it seems fitting also that he should really possess such powers, and that they should be used in cases of obvious necessity. But it is evident on a very cursory consideration that a public infirmary must ordinarily be an entirely suitable place for the retention of dead bodies awaiting inquiry, and also for the purposes of the actual inquiry itself. The principal witnesses are, no doubt, for the most part, on the spot, and there must be every conceivable facility for rendering the inquiry complete. One can imagine cases in which the removal of bodies from the infirmary to the mortuary may be desirable, but such cases must be very rare indeed. For the coroner to exercise his admitted power in cases where there is not the slightest necessity for the removal of bodies, and to put the infirmary authorities to inconvenience, and possibly to expense, seems to us to be the superfluity of silliness as well as of naughtiness. How very speedily a little common sense and decency of feeling would put an end to this ridiculous controversy. It is the coroner, obviously, who lacks the *savoir faire*.

**New Treatments, New Dangers.**

UNDER the heading of "Dangers of Antiseptic Midwifery," the *British Medical Journal* sets forth the results of Dr. Schräder's enquiries in this field of scientific investigation. Those results are sufficiently alarming. Antiseptic midwifery, according to this investigator, has sometimes killed patients who, but for such interference, would have recovered without any trouble more serious than a little feverishness. But until quite recently medical men have been under the conviction that, if they could be sure of anything at all, it was of the absolute value of antiseptic midwifery. To practice midwifery in any other way than antiseptically was to invite disaster and to prove oneself little better than a criminal. But now we must change all that, avers Dr. Schräder. The alternate "Yes" and "No" of modern medicine is distressingly confusing to the average medical mind. It may not appear to be serious to the hospital experimenter, who does not acknowledge a very painful responsibility for results so long as he is allowed to make his experiments in peace. But to the private practitioner, whose very existence may sometimes depend upon his being "up to date," the constant somersaults between "yes" and "no" present a tremendously harassing condition of things. There is an amusing side to the business, if amusement is ever compatible with such serious affairs as those of life and death. The student of our weekly medical literature who is old enough to compare some of the theories of to-day with those of twenty years ago, often asks as Pilate asked, "What is truth?" If he be a man of general reading the schoolman's problem of "How many angels can dance on the point of a needle?" will inevitably recur to him and provoke a smile. The amusing thing is that, however dogmatic and infallible the experimenter of to-day may be, his predecessor of twenty years ago, who took an exactly contrary view, was equally infallible. Take this single question of antiseptic midwifery as an illustrative example. But what does it all show? It shows that art is long, and that common sense and honesty are the most valuable of all mental endowments. That man is happy who has twenty years of careful and intelligent bedside experience to fall back upon. He trusts himself at the bedside, boldly follows out his own line of things, and cures his patients. We must change with the times, no doubt, but the man of capacity and courage changes as the master of the times, not as their weathercock.

